# Additional cognitive behavior therapy for persistent postural-perceptual dizziness: a meta-analysis

**DOI:** 10.1016/j.bjorl.2024.101393

**Published:** 2024-01-24

**Authors:** Jialin Zang, Mohan Zheng, Hongyuan Chu, Xu Yang

**Affiliations:** a Department of Neurology, Peking University Aerospace School of Clinical Medicine (Aerospace Center Hospital), Beijing, China; b Department of Pediatrics, Peking University First Hospital, Beijing, China

**Keywords:** Dizziness, Cognitive behavioral therapy, Dizziness handicap inventory

## Abstract

**Objective:**

To investigate whether additional Cognitive Behavior Therapy (CBT) combined with conventional therapy improves outcomes for patients with Persistent Postural-Perceptual Dizziness (PPPD) compared with conventional therapy alone.

**Methods:**

Two reviewers independently searched PubMed, Embase, Web of Science, Cochrane Library, and ClinicalTrials.gov for relevant Randomized Controlled Trials (RCTs) examining CBT for PPPD which were conducted and published in English from January 2002 to November 2022. RCTs reporting any indicators for assessing corresponding symptoms of PPPD were included, such as Dizziness Handicap Inventory (DHI), Hamilton Anxiety Scale (HAMA), Hamilton Depression Scale (HAMD), Hospital Anxiety and Depression Scale (HADS), and Patient Health Questionnaire-9 (PHQ-9). Two independent reviewers conducted extraction of relevant information and evaluation of risk of bias. The Cochrane Collaboration risk of bias tool version 1.0 was used to evaluate risks and assess the quality of the included studies, and Cochrane Review Manager 5.3 software (RevMan 5.3) was used to perform meta-analyses.

**Results:**

The results of six RCTs indicated that combining additional CBT with conventional therapy significantly improved outcomes for PPPD patients compared with conventional therapy alone, especially in DHI-Total scores (Mean Difference [MD = −8.17], 95% Confidence Interval [95% CI: −10.26, −6.09], *p* < 0.00001), HAMA scores (MD = −2.76, 95% CI: [−3.57, −1.94], *p* < 0.00001), GAD-7 scores (MD = −2.50, 95% CI [−3.29, −1.70], *p* < 0.00001), and PHQ-9 scores (MD = −2.29, 95% CI [−3.04, −1.55], *p* < 0.00001). Subgroup analysis revealed a significant benefit of additional CBT compared with conventional therapies alone, including Vestibular Rehabilitation Therapy (VRT) (MD = −8.70, 95% CI: [−12.17, −5.22], *p* < 0.00001), Selective Serotonin Reuptake Inhibitor (SSRI) (with controlled SSRI: MD = −10.70, 95% CI: [−14.97, −6.43], *p* < 0.00001), and VRT combined with SSRI (MD = −6.08, 95% CI [−9.49, −2.67], *p* = 0.0005) in DHI-Total scores.

**Conclusion:**

Additional CBT combined with conventional therapy may provide additional improvement for patients with PPPD compared with conventional therapy alone. However, more RCTs are needed to support and guide the application of CBT in treating PPPD.

**Level of evidence:**

I; Systematic review of RCTs.

## Highlights

• Cognitive Behavioral Therapy (CBT) has been widely used in patients with mild to moderate anxiety and depression.
• Additional CBT combined with conventional therapy may offer additional improvement to patients with PPPD.
• CBT could promote the improvement of symptoms and functional recovery, thereby producing therapeutic effects.

## Introduction

Persistent Postural-Perceptual Dizziness (PPPD) is a chronic functional vestibular disorder that is characterized by identifiable and unique sets of symptoms, comprising three main symptom clusters: dizziness, unsteadiness, and non-spinning vertigo that is present on most days for 3 or more months,^1^, ^2^ which may be exacerbated by upright posture, active or passive motion stimuli without regard to direction or position, and exposure to moving visual stimuli or complex visual patterns, and may be precipitated by conditions that cause vertigo, unsteadiness, dizziness, or problems with balance.^3^, ^4^ In tertiary dizziness centers, PPPD is the second most common diagnosis, accounting for approximately 15%–20% of cases.^5^ PPPD is often underestimated in community patients because related symptoms are easily overlooked, resulting in many patients not receiving early and accurate diagnosis and treatment.

Although PPPD is a new medical term, the phenomenon has long been recognized as a disorder of spatial orientation and motor sensory abnormalities. In the past 30 years, from the initial presentation of Phobic Positional Vertigo (PPV) to the subsequent presentation of Spatial Motion Discomfort (SMD), Visual Vertigo (VV), and Chronic Subjective Dizziness (CSD), the 2017 Bárány Association summarized and summarized the common issues related to PPV, SMD, VV, and CSD, whose commonalities form the basis of the diagnostic criteria for PPPD.^1^, ^3^ However, after searching in related fields, no Randomized Controlled Trials (RCTs) of PPV, SMD, or VV were found. As a result, six RCTs including patients with PPPD and CSD were included in the meta-analysis.

Although some debate remains, the most widely accepted putative mechanism of the pathogenesis of PPPD comprises a developmental process that involves functional, psychological, and structural disorders. The model of the mismatch between function and vestibular needs has been gradually accepted as explaining the probable mechanisms of PPPD. For a person without PPPD experiencing a precipitant vestibular crisis, medical event or acute psychological distress, acute adaptation including visual-somatosensory dependence, high-risk postural control strategies, and environmental vigilance may lead to neurotologic, medical, and behavioral recovery. After postural threats subside, the aforementioned strategies are typically abandoned. For patients with PPPD, however, additional predisposing factors such as neurotic temperament and pre-existing anxiety may cause behavioral comorbidity, including anxiety, and depressive and somatic symptom disorders. These factors can induce a vicious circle of maladaptation that is driven by perpetuating factors, including visual dependence, stiffened postural control, and decreased cortical integration, and provoking factors including upright posture, motion of self, and visual demands. Importantly, acute anxiety and body vigilance of PPPD patients can exacerbate the vicious circle mentioned above. Moreover, previous studies have indicated that damage of specific brain areas that are responsible for high-level spatial orientation, multi-sensory integration, and threat assessment plays a role in the mismatch of incoming and outgoing signals.^6^, ^7^

On the basis of current understanding of PPPD, a number of symptomatic treatments are recommended in patients with PPPD.^2^ These treatments are designed to reduce the patient’s anxiety traits to decrease the seizure of disease, or to improve the patient’s vestibular function to alleviate the symptoms of dizziness, unsteadiness, or vertigo. Vestibular Rehabilitation Therapy (VRT), which is generally used to treat vestibular diseases, aims to rebuild the balance system, focusing on high-risk postural control strategies, visual-somatosensory dependence, and environment vigilance.^8^, ^9^, ^10^ Additionally, Serotonin-Noradrenaline Reuptake Inhibitors (SNRIs) and selective Serotonin Reuptake iInhibitors (SSRIs) are commonly used in PPPD, mainly ealleviate mood disorders.^11^

Cognitive Behavioral Therapy (CBT) is a psychosocial intervention that has become the most widely used practice for improving mental health and is commonly used in patients with mild to moderate anxiety and depression.^3^ CBT is a counseling and treatment method that focuses on patients’ irrational cognitions and behaviors to improve negative emotions, and is a recommended treatment for PPPD.^12^, ^13^ CBT can provide talk therapy and stress management strategies to help patients understand and cope with their symptoms, focusing on the fear of falling, health anxiety, and avoidance behavior. CBT may benefit PPPD patients by reducing excessive physical alertness and fear of dizziness or falling down, and confronting anticipatory anxiety, and supplemental physical therapy can be performed to help patients become accustomed to exposure to reduce corresponding acute anxiety and body vigilance.^8^, ^10^ Because of its advantages, including a lack of side effects, low risk of potential harm, and convenience of administration, CBT has come to play an increasingly important role, and has received an increasing amount of attention from clinical physicians.

However, very few RCTs have studied the clinical efficacy of CBT in PPPD to date, and have typically had a small sample size, substantial variation in quality, and a lack of evidence-based evidence. Thus, a structured CBT intervention protocol that can strongly prove its efficiency and guide clinical practice has not yet been developed. To the best of our knowledge, no previous meta-analysis has statistically quantified the efficacy of CBT in PPPD. Therefore, the current meta-analysis aimed to collect and analyze available RCT data to provide a high-level evidence base regarding the efficacy of CBT and inform its application in the clinical field.

## Methods

### Search strategy

The meta-analysis was conducted according to the Preferred Reporting Items for Systematic Reviews and Meta-Analyses (PRISMA) statement and was previously registered with PROSPERO (nº CRD42022371200).

Two reviewers independently searched the PubMed, Embase, Web of Science, Cochrane Library, and ClinicalTrials.gov databases for relevant RCTs examining the use of CBT for patients with PPPD (details in Appendix), which were conducted and published in English from January 2002 to November 2022. The references of the included articles and relevant reviews were also manually searched and managed using EndNote X9. Studies were selected by screening the titles, abstracts, and content of the following inclusion and exclusion criteria by the two independent reviewers. Differences and disagreements were finally decided by the senior reviewer.

### Inclusion criteria

1) Patients: Patients of any age, sex, or nationality, diagnosed with PPPD, CSD, PPV, SMD, or VV on the basis of clinical characteristics and other examination methods.
2) Intervention: Patients in experimental groups were treated with CBT accompanied with corresponding conventional therapy in control groups.
3) Comparison: Patients in control groups were treated with conventional therapy, including VRT, SSRI, SNRI.
4) Outcome: Any indicators used to assess corresponding symptoms of PPPD, including vestibular symptoms, living quality, physical ability, functional balance, or emotional status. Preferred indicators in this field were as follows: Dizziness Handicap Inventory (DHI), Hamilton Anxiety Scale (HAMA), Hamilton Depression Scale (HAMD), Hospital Anxiety and Depression Scale (HADS), Patient Health Questionnaire-9 (PHQ-9), Generalized Anxiety Disorder Scale-7 (GAD-7), Visual Analog Scale.
5) Study design: RCT.

### Exclusion criteria

1) Animal research;
2) Patients not diagnosed with PPPD;
3) No single additional method of intervention;
4) Basic research, non-RCT, case report, review, systematic review, or meta-analysis;
5) No title or abstract available in English;
6) Incomplete studies without results.

### Data extraction

The relevant information and data provided in the eligible studies were extracted by two independent reviewers using a standardized data collection form, which comprised the author’s name, year of publication, condition of patients, treatment methods for the experimental and control groups, intervention time, home-based or supervised intervention, detailed data of outcome indicators, and other relevant information. A table of the RCT information is shown below.

### Study quality

Risks of bias assessment for studies included are shown in Table 1. The Cochrane Collaboration risk of bias tool version 1.0 (ROB 1.0) was used to evaluate corresponding risks to assess the quality of included studies in the meta-analysis.^14^ This tool mainly assesses bias from the random sequence generation, allocation concealment, blinding of participants and personnel, blinding of outcome assessment, incomplete outcome data, selective reporting, and other sources of bias. The two reviewers independently conducted the assessment process, in which differences in opinion were discussed to obtain consensus or decided by the senior reviewer.

**Table 1:** Risk of bias assessment for studies included.

Study	Random sequence generation	Allocation concealment	Blindinng of participants and personel	Blinding of outcome assessment	Incomplete outcome data	Selective reporting	Other sources of bias
Edelman 2012	Low risk	Low risk	High risk	Low risk	Low risk	Low risk	Low risk
Herdman 2022	Low risk	Low risk	High risk	Low risk	High risk	Low risk	Low risk
Qin 2016	Low risk	Low risk	High risk	Low risk	Low risk	Low risk	Low risk
Yu 2018	Low risk	Low risk	High risk	Low risk	Low risk	Low risk	Low risk
Yuan 2016	Low risk	Low risk	High risk	Low risk	High risk	Low risk	Low risk
Zhao 2022	Low risk	Low risk	High risk	Low risk	High risk	Low risk	Low risk

### Data analysis

Reviewers analyzed extracted data using Cochrane Review Manager 5.3 (RevMan 5.3) (https://revman.cochrane.org/). Indicator results of intervention and comparison groups were recorded and the outcomes (including DHI-Total, Functional, Emotional, Physical scores, HAMA scores, GAD-7 scores, and PHQ-9 scores) were then analyzed by group and subgroup using forest plots to obtain the Mean Differences (MD), 95% Confidence Intervals (95% CI), and *p-*values. The I^2^ statistic was used to quantify the magnitude of heterogeneity between studies,^15^ where I^2^ > 50% indicated high heterogeneity. If significant heterogeneity was not detected, a fixed-effects model would be adopted rather than a random-effects model. A sensitivity analysis was performed by excluding any study with a high risk of bias or using a fixed-effects model. Agreement between the models was assessed to ensure the reliability of the results. Publication bias was investigated visually using funnel plots in the meta-analysis.

## Results

### Data retrieval

**Figure 1:**
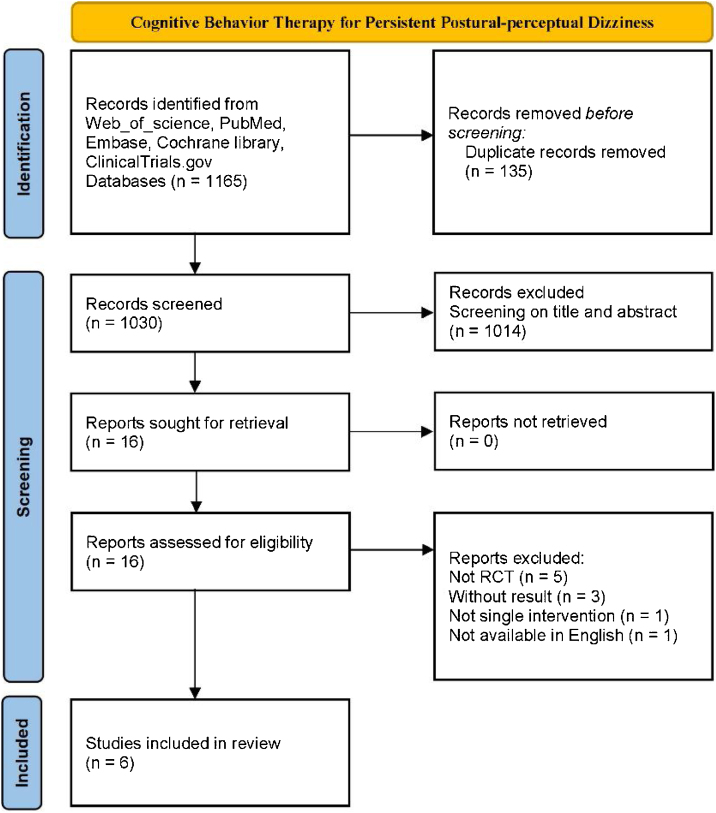
Flowchart diagram of included studies.

After screening the titles and abstracts of all 1030 studies, 16 studies that met the criteria were assessed for eligibility by further reviewing of the full text. Six RCT studies containing 416 participants were ultimately included in the review^12^, ^16^, ^17^, ^18^, ^19^, ^20^ (Fig. 1). Of the participants, 289 (69.5%) were female, and 127 (30.5%) were male. The average age of participants was 43.96 years, (experiment group: 43.89 years; control group: 43.72 years). Among the six studies included in the meta-analysis, three studies involved a total of 199 (47.8%) PPPD patients, and the other three studies involved 217 (52.2%) CSD patients. For the control group, 1 (16.7%) study used the method of delaying treatment (41 patients), 3 (50%) studies used a VRT group (198 patients), and 2 (33.3%) studies used an SSRI group (177 patients). In the experimental group, all studies tested the effects of additional CBT in comparison with a corresponding control group, 2 (33.3%) studies used a combination of supervised and home exercise, and 4 (66.7%) studies used supervised exercise. The specific characteristics of the included studies are shown in Table 2.

**Table 2:** Characteristics of studies included in the meta-analysis.

Study	Experiment Group	Control Group	Intervention time	Conducted	Intervention description	Control description	Type of study
Edelman 2012	20-subjective with Chronic Subjective Dizziness	21-subjective with Chronic Subjective Dizziness	3-weekly CBT sessions.	Supervised	Psychoeducation, behavioral experiments, exposure to feared stimuli, and attentional refocusing for 4-weeks	No treatment during RCT, commenced treatment 4-weeks later	RCT
Herdman 2022	19-subjective with Persistent Postural Perceptual Dizziness	20-subjective with Persistent Postural Perceptual Dizziness	6-sessions of INVEST for 4-months, initial session for 60-min, others for 30-min	Supervised	Transparency in communication with cognitive behavioral formulation and psychoeducation, normaling maladaptive postural strategies and attention allocation and relaxation techniques	6-sessions of individual VRT for 4-months including a range of general exercises and more specific adaptation, habituation, visual desensitization, static and dynamic balance exercises.	RCT
Qin 2016	30-subjective with Chronic Subjective Dizziness	30-subjective with Chronic Subjective Dizziness	8-weekly CBT + VRT sessions; one CBT session every 2-weeks for 8-weeks	Supervised and home exercise	Psychological support, health education, cognitive remediation training, exposure training, relaxation training	Balance function training, visual stimulation adaptation training, sports acclimatization training, conducted in both intervention and control group.	RCT
Yu 2018	46-subjective with Persistent Postural Perceptual Dizziness	45-subjective with Persistent Postural Perceptual Dizziness	2-CBT sessions per week for 8-weeks; sertraline every day for 8-weeks	Supervised	Earning the trust of patients; encouraging the patients to communicate with others; making patients expose and check the social factors that cause the PPPD, such as family, work, and social intercourse; making patients have a correct understanding of the occurrence, development, and treatment of PPPD.	Sertraline 50 mg/d ∼200 mg/d, conducted in both intervention and control group.	RCT
Yuan 2016	48-subjective with Chronic Subjective Dizziness	45-subjective with Chronic Subjective Dizziness	8-weekly CBT sessions, 3-VRT sessions every week for 8-weeks	supervised and home exercise	Psychological support, health education, cognitive assessment, relaxation training cognitive remediation training, behavioral training	Cawthorne-Cooksey, sports acclimatization training gaze stabilization training, conducted in both intervention and control group.	RCT
Zhao 2022	40-subjective with Persistent Postural Perceptual Dizziness	42-subjective with Persistent Postural Perceptual Dizziness	9-CBT sessions for 8-weeks	Supervised and home exercise	Disease acceptance, sleep improvement exercise exercises, dizziness emotional cognition, vestibular adaptation training, self-identification and observation, cognitive consolidation and relaxation exercises, emotional management functional activity-related training, exposure exercises, persistence and consolidation	SSRI (Escitalopram Oxalate) 5 mg/d, 10 mg/d after 5 d; Vestibular conditioning, static and dynamic balance training, alternative training, functional mobility related training, conducted in both intervention and control group.	RCT

### Heterogeneity analysis

The *I^2^* test showed there was low heterogeneity among all six studies included in the meta-analysis, which typically indicates there are few methodological heterogeneity issues, and that the corresponding results are credible.^15^

### Main outcome: meta-analysis of DHI-Total scores between groups receiving additional CBT and those receiving conventional therapy alone

**Figure 2:**
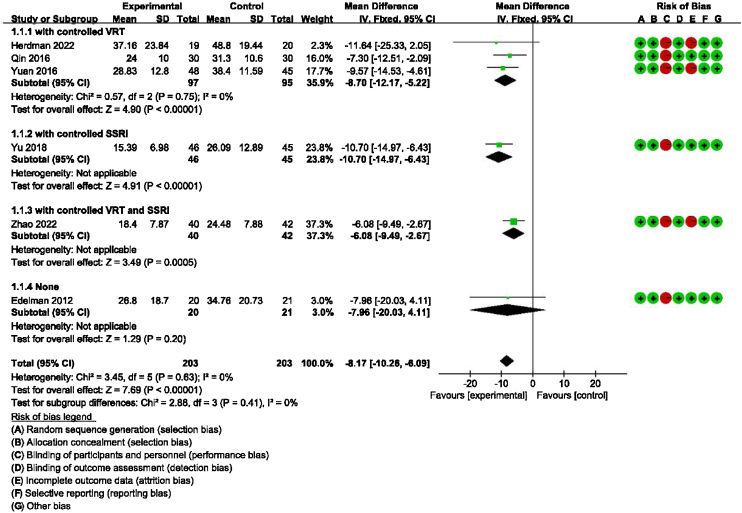
Forest plot for DHI-Total scores in groups with and without additional CBT.

Detailed data for DHI-Total scores of all six studies (406 participants) showed a significant benefit of additional CBT compared with conventional therapy alone (MD = −8.17, 95% CI: [−10.26, −6.09], *p* < 0.00001). The subgroup analysis also showed a significant benefit of additional CBT compared with VRT alone (MD = −8.70, 95% CI: [−12.17, −5.22], *p* < 0.00001), SSRI alone (MD = −10.70, 95% CI: [−14.97, −6.43], *p* < 0.00001), and VRT combined with SSRI (MD = −6.08, 95% CI: [−9.49, −2.67], *p* = 0.0005, Fig. 2).

### Main outcome: meta-analysis of DHI-functional, physical and emotional scores between groups receiving additional CBT and those receiving conventional therapy alone

**Figure 3:**
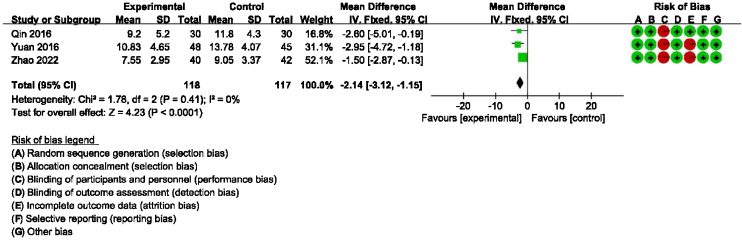
Forest plot for DHI-Functional scores in groups with and without additional CBT.

**Figure 4:**
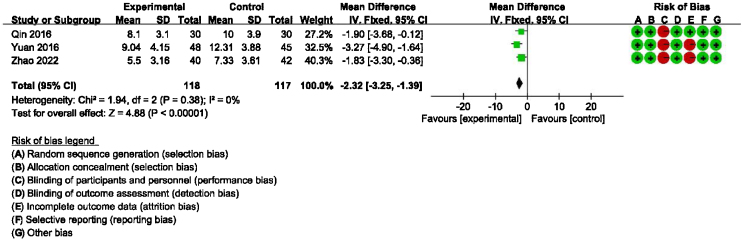
Forest plot for DHI-Physical scores in groups with and without additional CBT.

**Figure 5:**
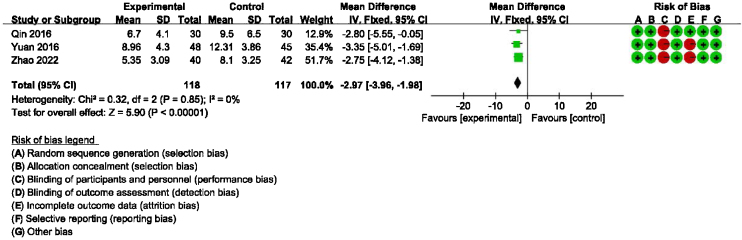
Forest plot for DHI-Emotional scores in groups with and without additional CBT.

Detailed data for DHI-Physical, Emotional and Functional scores from three studies (235 participants) showed a significant benefit of additional CBT compared with conventional therapy alone (DHI-P: MD = −2.32, 95% CI: [−3.25, −1.39], *p* < 0.00001; DHI-E: MD = −2.97, 95% CI: [−3.96, −1.98], *p* < 0.00001; DHI-F: MD = −2.14, 95% CI: [−3.12, −1.15], *p* < 0.0001, Figure 3, Figure 4, Figure 5).

### Secondary outcomes: meta-analysis of HAMA, GAD-7, PHQ-9 scores between groups receiving additional CBT and those receiving conventional therapy alone

**Figure 6:**
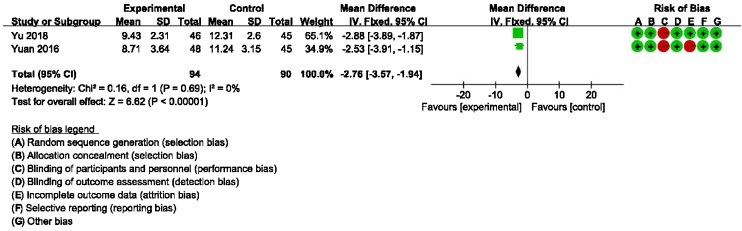
Forest plot for HAMA scores in groups with and without additional CBT.

**Figure 7:**
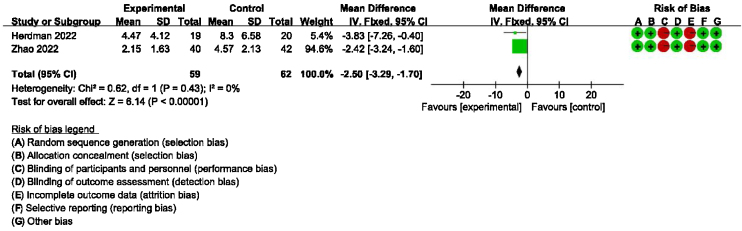
Forest plot for GAD-7 scores in groups with and without additional CBT.

**Figure 8:**
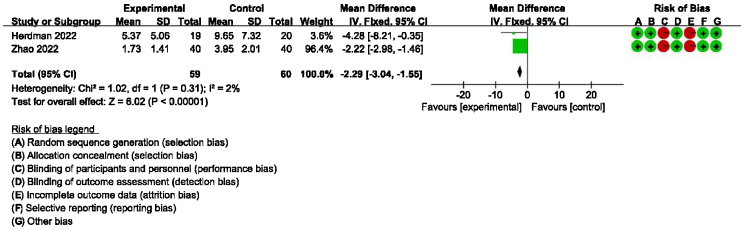
Forest plot for PHQ-9 scores in groups with and without additional CBT.

Detailed data for HAMA scores of two studies (184 participants) showed a significant benefit of additional CBT compared with conventional therapy alone (MD = −2.76, 95% CI: [−3.57, −1.94], *p* < 0.00001, Fig. 6). Detailed data for GAD-7 scores of two studies (121 participants) showed a significant benefit of additional CBT compared with conventional therapy alone (MD = −2.50, 95% CI: [−3.29, −1.70], *p* < 0.00001, Fig. 7). Detailed data for PHQ-9 scores of two studies (119 participants) showed a significant benefit of additional CBT compared with conventional therapy alone (MD = −2.29, 95% CI: [−3.04, −1.55], *p* < 0.00001, Fig. 8).

### Sensitivity analysis

Exclusion of any one of all 6 studies or using a random-effects model showed no difference in outcomes (MD [95% CI]: −9.42 [−12.05, −6.79], −7.39 [−9.77, −5.00]), which indicated that the results were not significantly affected by any individual study, or the chosen type of model.

### Publication bias

The funnel plots showed mild asymmetry, suggesting that the publication bias of the reviewed studies was mild and would not be likely to affect the results (Appendix Figs. 1‒7).

## Discussion

The current meta-analysis aimed to investigate the efficiency of additional CBT for patients with PPPD compared with conventional therapy alone. Because of the continuous optimization of the concept of PPPD, PPPD and its four subtypes (CSD, PPV, SMD, VV) were all intended to be incorporated in the meta-analysis. However, the literature search revealed no RCTs examining PPV, SMD, or VV. As a result, six RCTs examining patients with PPPD and CSD were included in the meta-analysis.

The selection of outcome indicators was based on the main symptom clusters and corresponding anxiety or depression. The six RCTs focused on different areas using different outcome indicators, resulting in only a few common indicators that could be examined in the meta-analysis. Finally, the DHI, which focuses on vestibular symptoms including dizziness, unsteadiness, and vertigo, as well as physical ability, functional balance, and emotional status, and the HAMA, GAD-7, and PHQ-9, which are focused on corresponding anxiety, were selected for use in the meta-analysis. Among these, the DHI, GAD-7, and PHQ-9 were self-rating scales, and HAMA was an examiner-rating scale.

After searching studies according to the search strategy, the relevant information, and data of the included RCTs were extracted for analysis, and ROB 1.0 was used to evaluate their corresponding risks to assess quality. The *I^2^* test showed low heterogeneity among all studies which suggested that the corresponding results were credible. As a result, a fixed-effects model was used.

DHI and anxiety- or depression-related scales were used to evaluate the efficacy of CBT in the meta-analysis. DHI is the most widely used scale for assessing the self-perceived handicapping effects of vestibular system disease, via self-assessment of disability associated with dizziness.^21^ Regarding the main outcome (DHI-Total scores), our meta-analysis of six studies suggested that additional CBT has significant benefits compared with conventional therapy alone. Additionally, the subgroup analysis showed significant benefits of additional CBT compared with VRT alone, SSRI alone, and VRT combined with SSRI. Moreover, regarding the main outcome of DHI-Functional, Physical and Emotional scores, four studies indicated that additional CBT has significant benefits compared with conventional therapy alone, and subgroup analysis showed significant benefits of additional CBT compared with VRT alone, SSRI alone, and VRT combined with SSRI. It should be noted that, in the subgroup analysis, although CBT was shown to be significantly effective compared with conventional therapies when used in combination, presenting CBT alone without combination with conventional therapy unexpectedly induced no additional significant improvement compared with a blank control group. However, caution should be exercised when drawing conclusions about whether CBT alone provides benefit in PPPD, because only one RCT included a CBT-only subgroup.^16^ This study was evaluated as having a high risk of bias, and the baseline was not balanced, with baseline DHI-Total scores in the control and experimental groups of 53.80 and 42.10, respectively, and outcome DHI-Total scores in the control and experimental groups of 26.80 and 34.76, respectively.^16^ Thus, more RCTs are warranted to explore whether CBT alone is effective without being used in combination with conventional therapy.

HAMA is the most widely-used examiner-rating scale, and is designed to measure the severity of anxiety neurosis,^22^ which may provide a measure of the change of anxiety before and after treatment, indicating that additional CBT provided effective relief for somatic anxiety and psychic anxiety in PPPD patients.^23^ The GAD-7 and PHQ-9 are commonly used self-rating scales for assessing patients’ anxiety, and have been shown to demonstrate excellent internal consistency.^24^, ^25^, ^26^ Regarding the secondary outcomes of HAMA, GAD-7, and PHQ-9 scores on the basis of our meta-analysis, additional CBT was associated with significant benefits compared with conventional therapy alone. However, it should be noted that only two studies for each of the HAMA, GAD-7, and PHQ-9 were included in the analysis.

Sensitivity and bias analysis were conducted. A sensitivity analysis in which each of the six studies was excluded and a random-effects model was used revealed no differences, indicating that the results of the meta-analysis were stable. The funnel plots indicated that the publication bias of the reviewed studies was mild and unlikely to affect the results, but also indicated that the number and sample size of studies were small, and few studies with negative results were identified.

The results indicated that additional CBT combined with conventional therapy could be beneficial for patients of PPPD in terms of physical ability, which reflects the severity of vertigo itself, functional balance, which reflects the degree of impact of vertigo on the patient’s mood, and emotional status, which reflects the degree of hindrance of vertigo to life, work, and social interaction, by reducing the patient’s anxiety level, which is an important factor in the vicious circle of maladaptation of PPPD.

The use of CBT in treating PPPD is primarily based on a theoretical framework in which anxiety is the core content of the psychophysiological model of the disease.^5^, ^27^, ^28^ Dizziness symptoms activate the human body’s internal threat response system, which increases the patient’s perception of motion stimuli. This can lead to PPPD and anxiety. When anxiety is induced, physical symptoms such as dizziness then occur, and mental and psychological mechanisms maintain these symptoms.^1^, ^5^ CBT focuses on the fear of falling, health anxiety, and avoidance behavior through talk therapy or stress management strategies, and can help patients understand and cope with their symptoms. Additionally, CBT may offer patients with PPPD benefits by reducing excessive physical alertness and the fear of dizziness or falling down, and confronting anticipatory anxiety, as well as supplemental physical therapy to help patients become accustomed to exposure to reduce corresponding acute anxiety and body vigilance.^8^, ^10^ CBT may be useful for correcting irrational cognition of patients with PPPD to enable cognitive reconstruction, and combining CBT and relaxation training may reduce or eliminate anxiety, fear and other negative emotions and corresponding physiological reactions, as well as promoting the improvement of symptoms and functional recovery, thereby producing therapeutic effects. Previous studies have reported that, when undergoing VRT, some patients feel very uncomfortable when exposed to eliciting stimuli, and exhibit aggravated anxiety reactions after treatment; techniques such as explanation, support, and behavioral relaxation using CBT may be useful in such situations.^3^, ^5^, ^27^ Thus, CBT may play a helpful role in providing timely and effective interventions to reduce emotional and psychological reactions in patients, enhance confidence, ensure smooth progress of rehabilitation, and improve curative effects.^5^

Overall, the current meta-analysis of multi-faceted questionnaire findings indicated that CBT significantly improved vestibular symptoms, mood, and social function. On the basis of an integrated analysis of RCT evidence, high-level recommendations can be made. The current findings suggest that it may be helpful for doctors to assess the subjective symptoms of patients with vertigo as a whole by counting the negative impact of vertigo on the patient’s body, emotional state, function and its severity. This approach may be conducive to the classification and screening of patients to determine individualized treatment plans, and evidence-based suggestions for improving the quality of life of patients with vertigo. However, more RCTs with larger sample sizes are still needed to further support the effectiveness of CBT for patients with PPPD.

### Limitations

Because of the continuous optimization of the concept of PPPD, there have been relatively few RCTs of standard PPPD treatment. Thus, various PPPD subtypes were included in the meta-analysis, possibly leading to clinical heterogeneity. Although the heterogeneity of the meta-analysis was 0%–2%, more studies with larger sample sizes are still needed to confirm the generalizability of the conclusion that additional CBT combined with traditional therapy is effective for treating PPPD. Although six RCTs were included in the current meta-analysis, the control groups of the included RCTs were diverse, and the subgroup analysis of individual conventional therapies lacked a sufficient number of RCTs to conduct an in-depth examination. Moreover, because different indicators of outcomes were used in different studies, there were few common indicators for analysis. Meanwhile, although DHI-Total scores were available in most included studies, the results of DHI-Physical, Emotional, and Functional scores were not reported or accessible in some RCTs. In addition, the details of the interventions examined in each RCT varied, limiting the ability to draw firm conclusions.

## Conclusions

The results of the current meta-analysis suggest that additional CBT combined with conventional therapy may provide significant benefits compared with conventional therapy alone for patients with PPPD. The meta-analysis revealed significant improvements in outcomes assessed using the DHI, HAMA, PHQ-9, and GAD-7. However, the limited number of RCTs prevented more definitive conclusions from being drawn, which indicates that more data are still needed to support and guide the application of CBT in the treatment of PPPD. With the rapidly growing international interest in advances in this field, there is substantial potential for CBT in the treatment of PPPD.

## Funding

This meta-analysis was supported by Natural Science Foundation of Beijing (Grant nº 7222237), on the mechanism of the central static compensation-related cerebellum-medial vestibular nucleus nerve pathway in Unilateral Peripheral Vestibular Disease (UPVD).

## Conflicts of interest

The authors declare no conflicts of interest.
