# Supplementary material for: Additional cognitive behavior therapy for persistent postural-perceptual dizziness: a meta-analysis
Source: Braz J Otorhinolaryngol. 2024 Jan 24;90(3):101393. doi: 10.1016/j.bjorl.2024.101393 (PMC10867767; doi:10.1016/j.bjorl.2024.101393)

BJORL-D-23-00240 _Supplementary Material

**Appendices** Supplement File Search Strategy.

| **Pubmed** |
| --- |
| (persistent postural perceptual dizziness [tiab] OR chronic subjective dizziness [tiab] OR phobic postural vertigo [tiab] OR space motion discomfort [tiab] OR visual vertigo [tiab] OR chronic dizziness[tiab]) AND (therapy [tiab] OR treatment [tiab] OR management [tiab] OR non-medication [tiab] OR physical therapy exercise [tiab] OR cognitive behavior therapy [tiab] OR cognitive behavioral therapy [tiab] OR cognitive behaviour therapy [tiab] OR cognitive behavioural therapy [tiab] OR CBT [tiab] OR psychotherapy [tiab] OR psychotherapy* intervention [tiab] OR correction of therapy [tiab] OR psychosomatic interplay [tw] OR self-controlled desensitization [tw] OR self-management [tw] OR patient education [tw] OR psychoeducation [tw] OR self-observation [tw] OR desensitizing exercises [tw] OR exposure therapy [tw] OR cognitive technique [tw] OR relaxation technique [tw] OR acceptance and commitment therapy [tw]) AND ((randomized controlled trial [pt] OR controlled clinical trial [pt] OR randomized [tiab] OR controlled [tiab] OR placebo [tiab] OR clinical trials as topic [mesh: noexp] OR randomly [tiab] OR trial [ti]) NOT (animals [mh] NOT humans [mh])) |
|  |
| **Web_of_science** |
| (TI=(persistent postural perceptual dizziness OR chronic subjective dizziness OR phobic postural vertigo OR space motion discomfort OR visual vertigo OR chronic dizziness ) OR AB=(persistent postural perceptual dizziness OR chronic subjective dizziness OR phobic postural vertigo OR space motion discomfort OR visual vertigo OR chronic dizziness)) AND (TI=(therapy OR treatment OR management OR non-medication OR physical therapy exercise OR cognitive behavior therapy OR cognitive behavioral therapy OR cognitive behaviour therapy OR cognitive behavioural therapy OR CBT OR psychotherapy OR psychotherapy* intervention OR correction of therapy) OR AB=(therapy OR treatment OR management OR non-medication OR physical therapy exercise OR cognitive behavior therapy OR cognitive behavioral therapy OR cognitive behaviour therapy OR cognitive behavioural therapy OR CBT OR psychotherapy OR psychotherapy* intervention OR correction of therapy) OR TS=(psychosomatic interplay OR self-controlled desensitization OR self-management OR patient education OR psychoeducation OR self-observation OR desensitizing exercises OR exposure therapy OR cognitive technique OR relaxation technique OR acceptance and commitment therapy)) AND (TI=(randomized controlled trial OR controlled clinical trial OR randomized OR controlled OR placebo OR clinical trials OR randomly OR trial) OR AB=(randomized controlled trial OR controlled clinical trial OR randomized OR controlled OR placebo OR clinical trials OR randomly OR trial)) |
|  |
| **Embase** |
| (‘persistent postural perceptual dizziness’:ab,ti OR ‘chronic subjective dizziness’:ab,ti OR ‘phobic postural vertigo’:ab,ti OR ‘space motion discomfort’:ab,ti OR ‘visual vertigo’:ab,ti OR ‘chronic dizziness’:ab,ti) AND ((‘therapy’:ab,ti) OR (‘treatment’:ab,ti) OR (‘management’:ab,ti) OR (‘non-medication’:ab,ti) OR (‘physical therapy exercise’:ab,ti) OR (‘cognitive behavior therapy’:ab,ti) OR (‘cognitive behavioral therapy’:ab,ti) OR (‘cognitive behaviour therapy’:ab,ti) OR (‘cognitive behavioural therapy’:ab,ti) OR (‘CBT’:ab,ti) OR (‘psychotherapy’:ab,ti) OR (psychotherapy* intervention:ab,ti) OR (‘correction of therapy’:ab,ti) OR ‘psychosomatic interplay’/exp OR ‘self-controlled desensitization’/exp OR ‘self-management’/exp OR ‘patient education’/exp OR ‘psychoeducation’/exp OR ‘self-observation’/exp OR ‘desensitizing exercises’/exp OR ‘exposure therapy’/exp OR ‘cognitive technique’/exp OR ‘relaxation technique’/exp OR ‘acceptance and commitment therapy’/exp) AND ('crossover procedure':de OR 'double-blind procedure':de OR 'randomized controlled trial':de OR 'single-blind procedure':de OR (random* OR control* OR crossover* OR cross NEXT/1 over* OR placebo* OR doubl* NEAR/1 blind* OR singl* NEAR/1 blind* OR assign* OR allocat* OR volunteer*):de,ab,ti) AND ([2002-2022]/py) |
|  |
| **Cochrane library** |
| (((persistent postural perceptual dizziness OR chronic subjective dizziness OR phobic postural vertigo OR space motion discomfort OR visual vertigo OR chronic dizziness):ti) AND ((therapy OR treatment OR management OR non-medication OR physical therapy exercise OR cognitive behavior therapy OR cognitive behavioral therapy OR cognitive behaviour therapy OR cognitive behavioural therapy OR CBT OR psychotherapy OR psychotherapy* intervention OR correction of therapy OR psychosomatic interplay OR self-controlled desensitization OR self-management OR patient education OR psychoeducation OR self-observation OR desensitizing exercises OR exposure therapy OR cognitive technique OR relaxation technique OR acceptance and commitment therapy OR psychosomatic interplay OR self-controlled desensitization OR self-management OR patient education OR psychoeducation OR self-observation OR desensitizing exercises OR exposure therapy OR cognitive technique OR relaxation technique OR acceptance and commitment therapy):ti) AND (Between Jan 2002 and Nov 2022)) |
|  |
| **ClinicalTrials.gov** |
| **Condition or disease:** |
| Persistent postural perceptual dizziness OR chronic subjective dizziness OR phobic postural vertigo OR space motion discomfort OR visual vertigo |
|  |
| **Other terms:** |
| Therapy OR treatment OR management OR non-medication OR physical therapy exercise OR cognitive behavior therapy OR cognitive behavioral therapy OR cognitive behaviour therapy OR cognitive behavioural therapy OR CBT OR psychotherapy OR psychotherapy* intervention OR correction of therapy OR psychosomatic interplay OR self-controlled desensitization OR self-management OR patient education OR psychoeducation OR self-observation OR desensitizing exercises OR exposure therapy OR cognitive technique OR relaxation technique OR acceptance and commitment therapy OR psychosomatic interplay OR self-controlled desensitization OR self-management OR patient education OR psychoeducation OR self-observation OR desensitizing exercises OR exposure therapy OR cognitive technique OR relaxation technique OR acceptance and commitment therapy |
|  |
| **Study type:** |
| Interventional study (clinical trial) |
|  |
| **Study Results:** |
| Studies with results |

**Supplemental Figure 1** Funnel plot of DHI-Total scores of PPPD patients after conventional therapy plus additional CBT compared with conventional therapy alone. DHI, Dizziness Handicap Inventory; PPPD, Persistent Postural-Perceptual Dizziness; CBT, Cognitive Behavior Therapy.


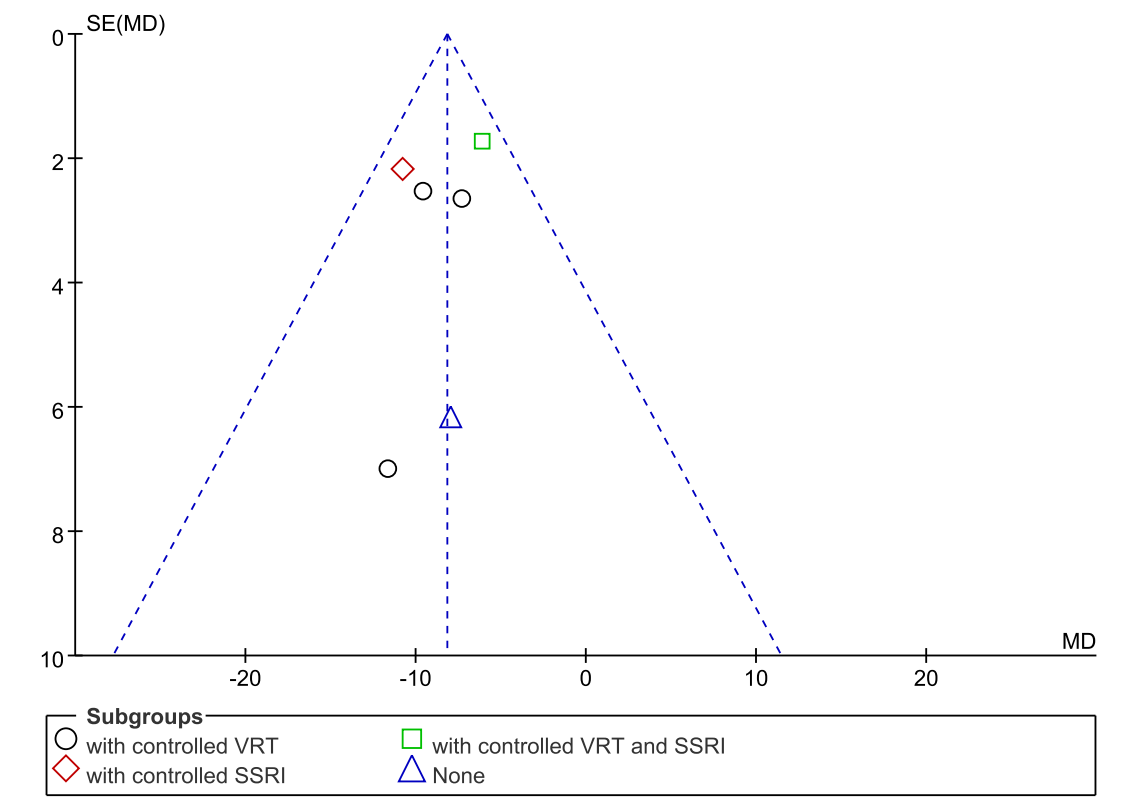


**Supplemental Figure 2** Funnel plot of DHI-Functional scores of PPPD patients after conventional therapy plus additional CBT compared with conventional therapy alone. DHI, Dizziness Handicap Inventory; PPPD, Persistent Postural-Perceptual Dizziness; CBT, Cognitive Behavior Therapy.


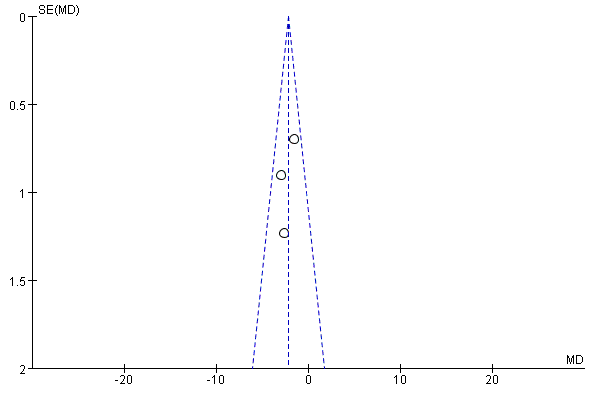


**Supplemental Figure 3** Funnel plot of DHI-Physical scores of PPPD patients after conventional therapy plus additional CBT compared with conventional therapy alone. DHI, Dizziness Handicap Inventory; PPPD, Persistent Postural-Perceptual Dizziness; CBT, Cognitive Behavior Therapy.


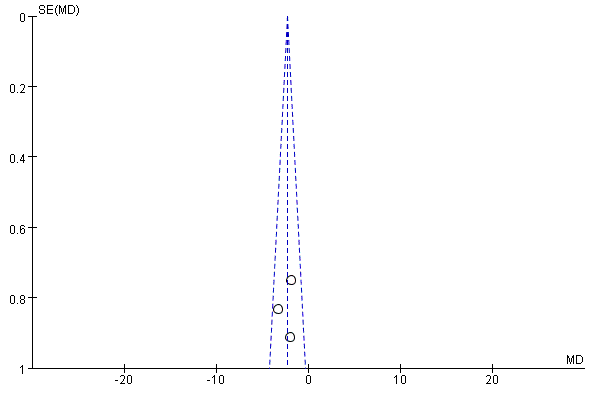


**Supplemental Figure 4** Funnel plot of DHI-Emotional scores of PPPD patients after conventional therapy plus additional CBT compared with conventional therapy alone. DHI, Dizziness Handicap Inventory; PPPD, Persistent Postural-Perceptual Dizziness; CBT, Cognitive Behavior Therapy.


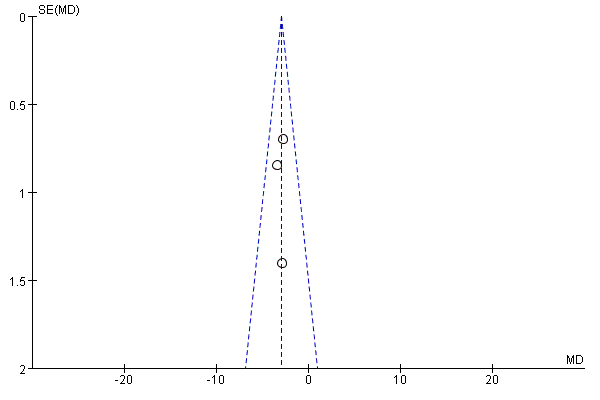


**Supplemental Figure 5** Funnel plot of HAMA scores of PPPD patients after conventional therapy plus additional CBT compared with conventional therapy alone. HAMA, Hamilton Anxiety Scale; PPPD, Persistent Postural-Perceptual Dizziness; CBT, Cognitive Behavior Therapy.


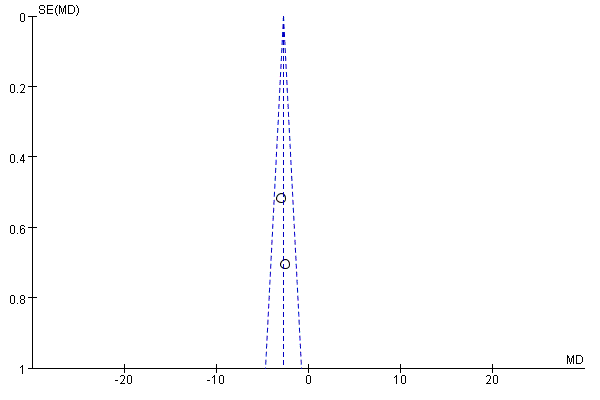


**Supplemental Figure 6** Funnel plot of GAD-7 scores of PPPD patients after conventional therapy plus additional CBT compared with conventional therapy alone. GAD-7, Generalized Anxiety Disorder Scale-7; PPPD, persistent postural-perceptual dizziness; CBT, cognitive behavior therapy.


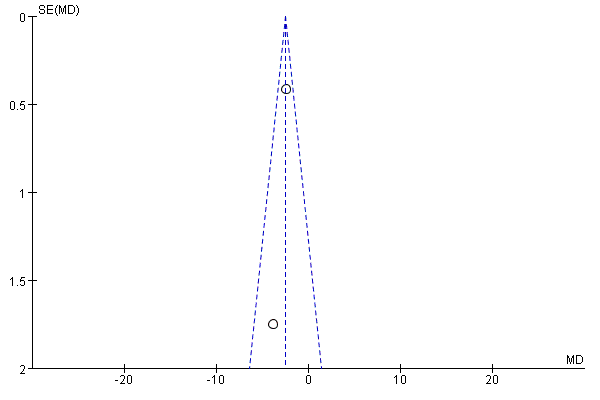


**Supplemental Figure 7** Funnel plot of PHQ-9 scores of PPPD patients after conventional therapy plus additional CBT compared with conventional therapy alone. PHQ-9, Patient Health Questionnaire-9; PPPD, Persistent Postural-Perceptual Dizziness; CBT, Cognitive Behavior Therapy.


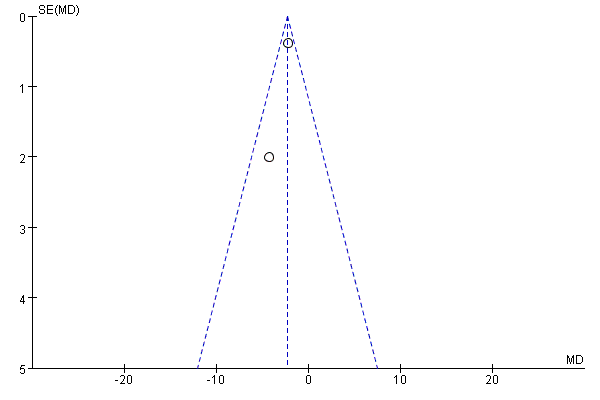

Supplement: Supplementary file 1 [file mmc1.docx]
